# Effects of rTMS over the contralateral M1 combined with NMES on pain and functional mobility in primary frozen shoulder: study protocol for a 4-week double-blind, randomized, sham-controlled trial

**DOI:** 10.3389/fneur.2025.1722035

**Published:** 2026-02-16

**Authors:** Huanxia Zhou, Enbang Zhang, Minghui Lai, Peige Wang, Shiyi Zhou, Yifan Wang, Yiming Xie, Yongfang Zhao

**Affiliations:** 1 Department of Rehabilitation Medical Center, Seventh People’s Hospital of Shanghai University of Traditional Chinese Medicine, Shanghai, China; 2 Department of Radiology, Seventh People's Hospital of Shanghai University of Traditional Chinese Medicine, Shanghai, China; 3 Shi’s Center of Orthopedics and Traumatology, Shuguang Hospital Affiliated to Shanghai University of Traditional Chinese Medicine, Shanghai, China; 4 Institute of Traumatology and Orthopedics, Shanghai Academy of Traditional Chinese Medicine, Shanghai, China; 5 School of Acupuncture-Moxibustion and Tuina, Shanghai University of Traditional Chinese Medicine, Shanghai, China; 6 School of Exercise and health, Shanghai University of Sport, Shanghai, China

**Keywords:** frozen shoulder, function, neuroplasticity, NMES, pain, rTMS

## Abstract

**Background:**

Repetitive transcranial magnetic stimulation (rTMS) with low and high frequency as well as neuromuscular electrical stimulation (NMES) have been proved efficacy, respectively, on pain and dysfunction of frozen shoulder (FS). Evidence suggests that the integration of central neuromodulation and peripheral stimulation techniques, establishing a closed-loop neural circuitry, yields superior therapeutic outcomes compared to isolated rehabilitation modalities. Therefore, the purpose of this study is to evaluate the clinical effectiveness and neuro-biomechanism of combining rTMS and NMES in alleviating pain and motor deficit of primary FS patients.

**Methods:**

This will be an assessor and patients blinded, sham controlled and randomized controlled clinical trial involving a 4-week intervention and a 6-month follow-up. 117 people with FS will be equally allocated to the sham-rTMS + NMES group (Group A), low frequency (LF)-rTMS + NMES group (Group B) and high frequency (HF)-rTMS+ NMES group (Group C) through stratified randomization. Interventions will be provided in 5 sessions per week, with a total of 20 sessions. The primary outcome measurements will be the score of Constant-Murley. The secondary outcome measurements will include polysomnography, quality of life (QOL) by SF-36, muscle biomechanics by surface electromyography (sEMG), motor evoked potential (MEP), brain neuroplasticity by magnetic resonance imaging (MRI). Evaluations will be performed at six time points, including at baseline, 2 weeks and 4 weeks from the start of treatment, and 1 month, 3 months and 6 months following the end of treatment. Two-way analysis of variance with repeated measures will be applied to examine the main effects of the group, the time and group-time interaction effects for all outcomes.

**Discussion:**

The study will be the first protocol to demonstrate the effectiveness of rTMS with/without NMES for improving shoulder function and pain management after FS and compare the efficacy of LF-rTMS and HF-rTMS. The results of the study may guide the design of more effective treatment methods for FS rehabilitation.

**Clinical trial registration:**

ChiCTR2500098406.

## Introduction

Frozen shoulder (FS), also known as adhesive capsulitis, is a persistent musculoskeletal disorder that manifests as severe insidious onset pain and functional constraint (1). Primary FS cause a progressive and excruciating loss of shoulder active and passive range of motion(ROM) without a known cause (2). FS affects 8% of men and 10% of women worldwide, with a lifetime frequency of 2 to 5% in the fifth and sixth decades. Instead of treating FS as self-limiting and proceeding through three overlapping stages without supervised intervention, evidence-based physiotherapy guidelines classify it as ‘pain predominant’ or ‘stiffness predominant’ (3). Between 7 and 50% of FS patients do not achieve normal ROM and endure symptoms for years with 6% lasting over 7 years (4, 5). Given that FS has proven resistant to treatment, exploration of emerging treatments is warranted (6).

The primary mechanism of shoulder pain in FS was seen as a peripheral nociception, characterized by elevated pro-inflammatory cytokines and neuroimmune system activation (7, 8). However, the pathophysiology of FS is intricate, with central pain pathways also playing a crucial role in patients with FS (9). FS patients exhibit more extensive pain regions, increased allodynia, hyperalgesia, catastrophizing, and central sensitization for pain in comparison to healthy individuals (10, 11). As the FS remains, the central nervous system may undergo potential neuroplastic changes that cause regional functional abnormalities (12), modifications in the pain modulation pathway (13) and network reorganizations (14, 15). These changes indicate a link between functional and morphological brain changes and neuroplasticity and neurobiological mechanisms generated by pain, representing cerebral cortex functional remodeling (16), similar to other chronic pain conditions (17, 18). Shoulder pain of FS is a continuous state, so FS patients both encompassing daytime pain and result in physical dysfunction and activity limitations and associated with risk of impaired sleep efficiency, length, and quality due to nocturnal shoulder pain and 34% of FS patients experience sleep disturbance and emotional disorders (19, 20, 21). The interplay of shoulder pain and sleep disturbance may exacerbate behavioral and biological quality of life (QoL), shoulder impairment, and daily activities.

Neuromuscular electrical stimulation (NMES) facilitates in blood circulation, muscle strength enhancement, tissue repair and analgesia by stimulating the selected muscles to induce muscle contraction following the depolarization of motor neurons (22, 23). It has discovered that stimulating the interscapular musculature, deltoid, triceps, and wrist extensors during a reaching task enhanced task performance by augmenting the ROM in active shoulder flexion and elbow extension (24). Researchers found that the cortical excitability was enhanced during motor activities subsequent to a brief administration of NMES combined with volitional activity in the upper extremity (25). Furthermore, a study involving 25 healthy participants revealed that NMES with voluntary movement of the stimulated muscles enhanced cortical excitability (26). The advantageous adaptation in the cerebral cortex may happen in neuroplasticity and functional modifications through NMES.

The maladaptive neuroplastic alterations are linked to FS, suggesting that treatment focused on reversing these changes is necessary (27). Repetitive transcranial magnetic stimulation (rTMS) is a non-invasive neuromodulation technique works by generating rapidly changing magnetic fields in specific regions of the cerebral cortex, which induce neuronal depolarization. This process can modulate neuronal excitability and synaptic plasticity in the underlying brain structures to restore cortical networks and may serve as suitable management alternatives for individuals with FS. The primary motor cortex (M1), located in the precentral gyrus of the frontal lobe is traditionally considered the brain region responsible for controlling voluntary movements of the contralateral limbs, however, recent studies have revealed that M1 also demonstrates neuroplasticity in relation to pain. The M1 neuronal projections to the periaqueductal gray (PAG) circuit encode information regarding sensory hypersensitivity associated with pain. M1 neurons can also project to tertiary neural circuits that include the mediodorsal thalamus and nucleus accumbent, recording the negative emotional dimensions of pain (28). The recent systematic review has also demonstrated that M1-rTMS can effectively reduce neuropathic pain (29), with M1 serving as the principal stimulation target for rTMS-induced analgesia. Multiple experimental studies have demonstrated that M1-rTMS possesses enhanced efficacy in reducing pain intensity, markedly diminishing the perceived severity of induced pain (30). The effects of rTMS at different frequencies on analgesia and cortical excitability differs: high-frequency (≥5 Hz) rTMS enhances cortical excitability, whereas low-frequency (≤1 Hz) rTMS diminishes cortical excitability. Both high and low frequencies may elicit analgesic effects (31). But the exact neural processes by which cortical plasticity contributes to FS remain unidentified.

If peripheral and cortical alterations are associated with FS, then rTMS and NMES training may be beneficial in treating this ailment for which effective treatment remains elusive. Therefore, the present protocol will be designed for a patient and assessor blinded, sham-controlled, randomized clinical trial, which will include 3 groups: Sham rTMS +NMES group (Group A), the LF-rTMS+NMES group (Group B) and the HF- rTMS+NMES group (Group C) and. The purposes of this protocol are as follows:

Aim 1: To evaluate the efficacy of NMES combined with rTMS over the contralateral M1 area in reducing pain intensity and improving functional ability in patients with FS. And to compare the efficacy of LF-rTMS+NMES and HF-rTMS+NMES in patients with FS.

Aim 2: To investigate the correlation between the clinical efficacy and neuro-biomechanism of cortical alternation in patients with FS after conducting systematic evaluations both before and after treatment.

Aim 3: Based on this study protocol and its anticipated outcomes, clinicians can develop innovative management strategies for pain and motor function in patients with FS, aiming to achieve effective therapeutic results.

## Methods and analysis

### Study design

**Figure 1:**
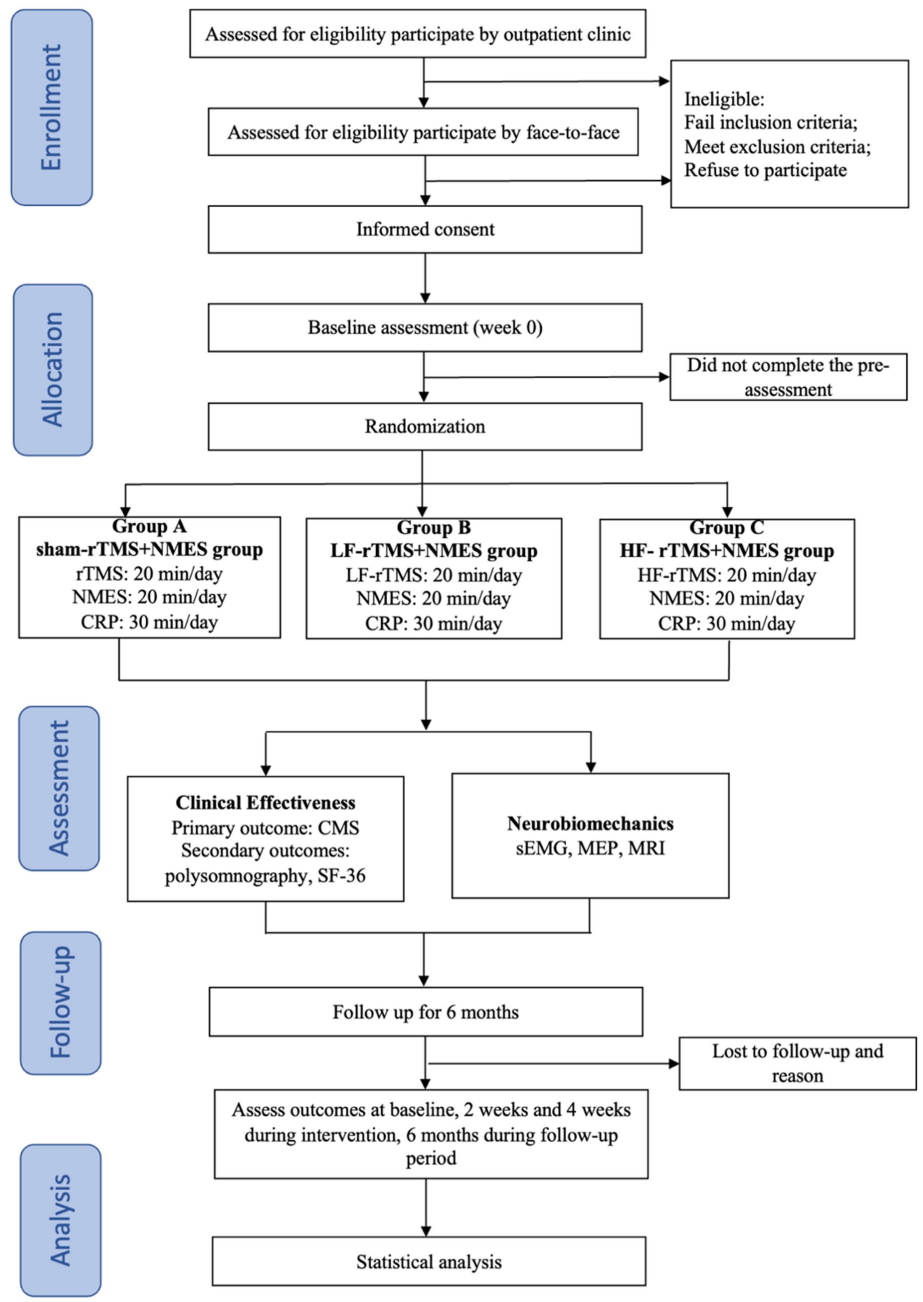
A brief flowchart of the entire study. FS, Frozen shoulder; CRP, conventional rehabilitation program; NMES, neuromuscular electrical stimulation; LF-rTMS, low-frequency repetitive transcranial magnetic stimulation; HF-rTMS, high-frequency repetitive transcranial magnetic stimulation; SF-36, 36-item short-form health survey; sEMG, surface electromyography; MEP, motor evoked potential; MRI, magnetic resonance imaging.

This will be an assessor and patients blinded, sham-controlled, randomized controlled clinical trial (RCT) involving a 4-week intervention and 6-month follow-up. A total of 117 patients with FS will be recruited and randomly assigned to the sham rTMS +NMES group (Group A), LF-rTMS+NMES group (Group B) and HF-rTMS+NMES group (Group C) at a ratio of 1:1:1. Figure 1 depicts a concise flowchart of the entire study, and Table 1 provides the schedule of events. The study protocol (2025-7th-HIRB-042) was approved by the Ethics Committee of the Seventh People’s Hospital of Shanghai University of Traditional Chinese Medicine and registered in the Chinese Clinical Trial Registry (ChiCTR2500098406).

**Table 1:** Schedule of enrolment, intervention and assessments.

Study period							
Timepoint	Screening	Baseline	Treatment		Follow- up		
	-1 month	0	2 weeks	4 weeks	1 month	3 months	6 months
Enrolment							
Eligibility screen	✓						
Informed consent	✓						
Demographic information	✓						
Descriptive information		✓					
Randomisation		✓					
Allocation		✓					
Interventions							
Group A		↔					
Group B		↔					
Group C		↔					
Assessments							
CMS		✓	✓	✓	✓	✓	✓
polysomnography		✓	✓	✓	✓	✓	✓
SF-36		✓	✓	✓	✓	✓	✓
sEMG		✓	✓	✓	✓	✓	✓
MEP		✓	✓	✓	✓	✓	✓
GMWM/FC/subcortical nuclei volumes cortical thickness (MRI)		✓		✓			✓
Acceptability assessments							
Referral rate				✓			✓
Retention rate				✓			✓
Drop-out rate				✓			✓
Safety assessments							
Adverse event occur rate				✓			✓
Summary the study	s						✓

“✓“means things will be done. CMS, Constant-Murley Score; SF-36, 36-Item Short Form Survey; sEMG, surface Electromyography; MEP, motor evoked potential; GM, grey matter density; WM, white matter; FC, functional connectivity; MRI, magnetic resonance imaging.

### Sample size calculation

The G*power software (version 3.1) was utilized to determine the minimal sample size necessary to identify a significant between-group difference in the present research. A systematic review and meta-analysis indicated that the pain and function are the primary outcome (32) with an effect size of 0.25 for CMS in detecting changes or improvements in pain and function scores in patients with FS (33). Based on a prior two-way repeated analysis of variance (ANOVA) F test, with a power of 0.80, an effect size of 0.25, three groups, six measurements, correlation among repeated measures of 0.5, and an alpha level of 0.05, 93 participants were required for this study. To account for a conservative drop-out rate of 20%, the final sample size will therefore be 39 participants in each group, for a total of 117 participants.

### Selection of subjects

#### Inclusion criteria

1. Patients aged ≥18 years old with unilateral shoulder pain.
2. A minimun 25% reduction in the ROM in at least two planes compared to the unaffected shoulder (34).
3. Patients with a duration of pain ≤ 9 months and a visual analogue scale (VAS) for shoulder pain ≥ 3.
4. Normal shoulder X-rays.

#### Exclusion criteria

1. Patients with secondary FS and additional shoulder conditions (e.g., shoulder joint deformity, fractures, dislocations, arthritis, cervical radiculopathy, and previous shoulder surgery, rotator cuff injuries or subacromial impingement syndrome, calcified tendinitis, and osteoarthritis).
2. Other shoulder pain secondary to conditions such as fibromyalgia, rheumatic diseases, and trauma.
3. Patients who received an intra-articular corticosteroid injection in the affected side within the preceding 3 months.
4. Patients diagnosed with bilateral FS.
5. Other comorbidities (i.e., cardiovascular, neurological, hepatic or renal disorders, malignant tumors, high fever, infectious diseases and cognitive impairments, etc).
6. Individuals unable to finish the MRI scan.
7. Patients with cognitive dysfunction, mental illness or inability to understand and cooperate with the investigators or provide informed consent.

### Setting and recruitment

A total of 117 individuals who comply with the inclusion criteria will be recruited from Seventh People’s Hospital of Shanghai University of Traditional Chinese Medicine and neighboring communities using flyers, posters, and referrals from an orthopaedician, physical therapist or occupational therapist. The participants will be invited to undergo an in-person examination and evaluation to guarantee that they satisfy the inclusion criteria. In addition, we will inform all participants of the study’s purpose, methodology, prospective benefits and the principle of voluntary participation. Recruitment began on 1 September 2025 and will continue until 117 individuals are enrolled.

### Randomization, allocation concealment and blinding

Each eligible participant will be associated with a randomly generated numerical code generated by SPSS software (IBM Corp. Released 2013. IBM SPSS Statistics for Windows, Version 29.0. Armonk, NY: IBM Corp.). To minimize any potential bias, participants will be randomly allocated to 3 groups according to pain intensity: mild pain (VAS 3–4), moderate pain (VAS 5–6), and severe pain (VAS 7–10) as variables (35, 36). Participants form each group will be randomly divided into groups A, B and C; thereafter, all groups A, B and C will be consolidated to create new groups (intervention group A, intervention group B and control group C) to minimize the bias of the results. The outcomes of the participants grouping will be sealed in opaque envelopes by an independent researcher and disclosed only at the time of allocation. Furthermore, therapists with equivalent qualifications, responsible for administering rTMS, NMES, and conventional rehabilitation programs, will likewise adhere to randomization and be randomly allocated to one of the groups. The visibility of the rTMS intervention prevents the operators from being blinded to the allocation of the intervention. Consequently, blinding will be implemented for both patients and assessors, as well as statisticians responsible for data collection and final statistical analysis in this study, to avoid any potential implementation and measurement bias.

### Intervention methods

The intervention will be administered at the rehabilitation medical center of the Seventh People’s Hospital of Shanghai University of Traditional Chinese Medicine. All participants will receive the conventional rehabilitation program (CRP) recommended by the FS rehabilitation guidelines (37), which will include low-high grade joint mobilization, stretching exercise, passive and active limb movements, muscle strengthening, and self ROM exercises like different directions reaching, grasping and daily actions that are motivational and appropriate for the participants. Based on the CRP (30 min), control group C will receive Sham rTMS (20 min) + NMES (20 min); Intervention group A will receive LF-rTMS (20 min) + NMES (20 min); and Intervention group B will receive HF-rTMS (20 min) + NMES (20 min). The intervention for the three groups will be provided for 20 sessions (5 sessions per week, 4 weeks), each lasting 70 min.

### Neuromuscular electrical stimulation (NMES)

**Figure 2:**
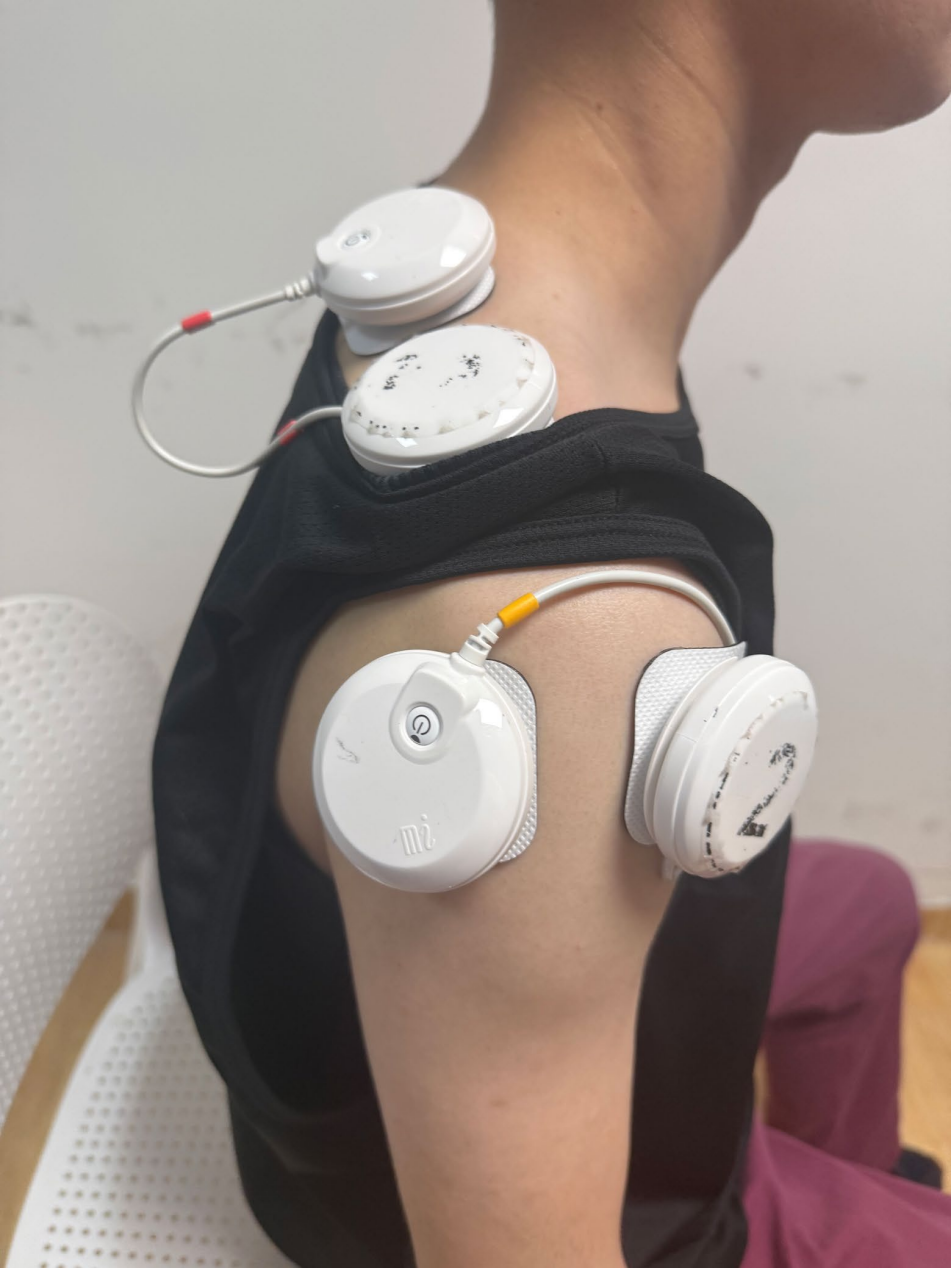
NMES treatment for FS patients.

NMES (1–150 Hz and pulse width30-400 μs, the Chattanooga Wireless Pro 4, DJO FRANCE SAS) is specifically created for people requiring pain management and muscle strengthening, among other applications. The surface electrodes will be placed near the motor points of upper trapezius, supraspinatus, and deltoids (anterior, middle and posterior bundles) (Figure 2). Prior research indicated that eliciting contraction of the upper trapezius aids in stabilizing and elevating the scapula, thereby leading to external rotation of the scapula with the acromioclavicular joint serving as the pivot (38). The supraspinatus stabilizes the humerus within the glenoid fossa and coordinates the deltoids during shoulder abduction. The contraction of the anterior, middle and posterior deltoids facilitated the shoulder flexion, abduction and extension. Therefore, by moving the surface electrode (area = 2 × 2 cm^2^) on the skin above these target muscle bellies until a site is identified where minimum current elicits a visible muscle contraction without discomfort (39), typically in the range of tens of milliamps (between 20 mA and 50 mA). In stimulation therapy, the stimulator executed a cycle every 30 s, comprising 5 s for ramp-up, 10 s at peak stimulation, 5 s for ramp-down, and 10 s of no stimulation (40).

### Repetitive transcranial magnetic stimulation (rTMS)

**Figure 3:**
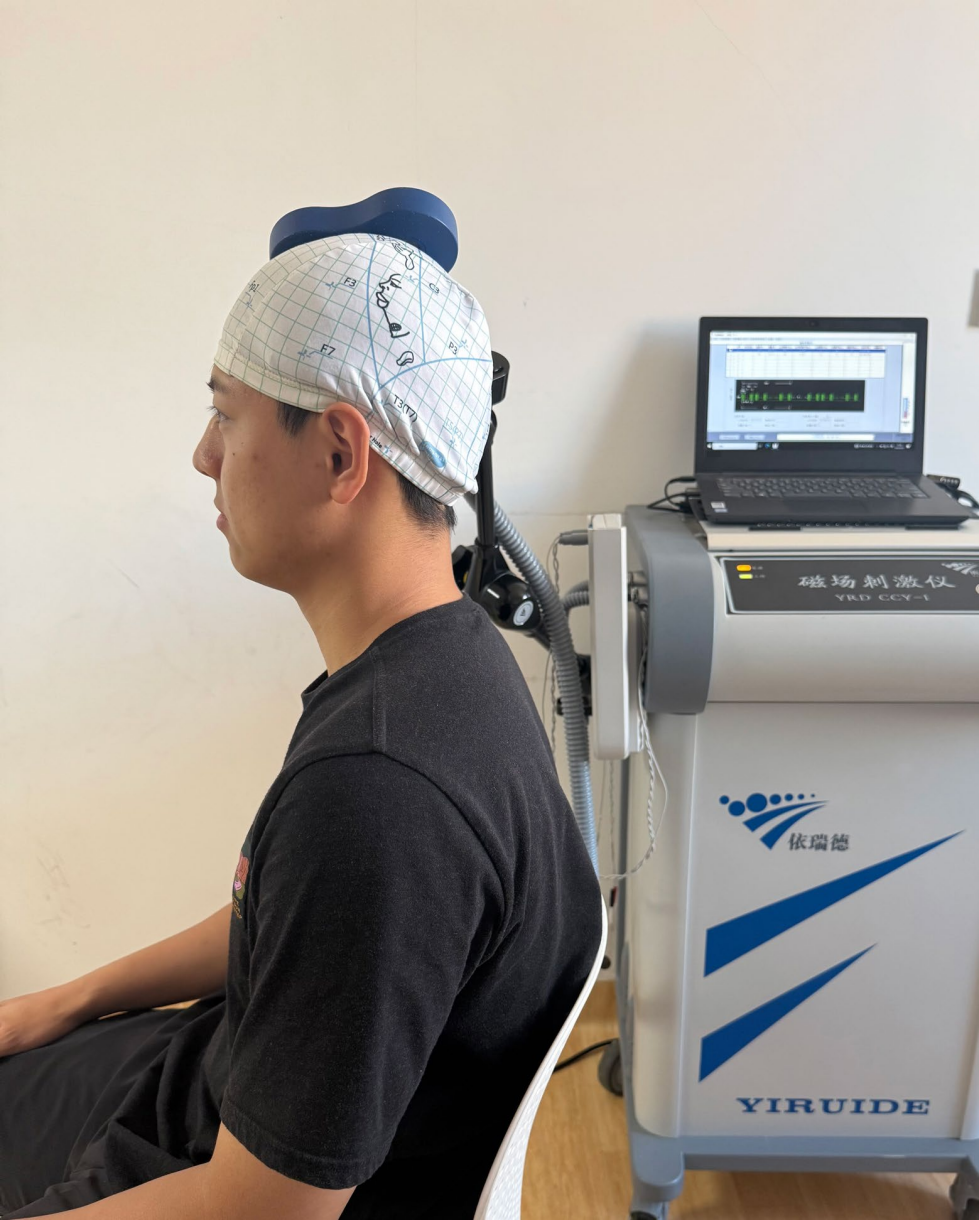
The rTMS measurement and intervention were performed on FS patients.

The rTMS will use a Super-Rapid Magstim Stimulator (The Magstim Co., Whitland, UK) equipped with a figure-of-8 coil. As the two rings converge, the electric field peaks at its center (hot spot), stimulating a more accurately defined area (41). All the rTMS program will follow the International Federation of Clinical Neurophysiology guideline (42). A total of 20 rTMS sessions were performed over 4 weeks, with 5 sessions on 5 consecutive workdays and a 2-day maintenance break. Each rTMS session consisted of 30 trains of TMS pulses administered at 1HZ(LF-rTMS) or 10 Hz(HF-rTMS) for 10 s (100 pulses/train) with a 20s intertrain interval, resulting in 4,000 pulses per session for a total duration of 20 min (43). The target of the motor cortical region (M1) is at the contralateral hemisphere of the affected shoulder. The stimulation intensity will be set at 80% of the resting motor threshold, which is the minimum intensity that elicits an electromyographic response ≥50 μV in the first dorsal interosseous (FDI) of the hand contralateral to the stimulated hemisphere in at least 5 out of 10 trials. In this measurement, the figure-of-8 coil (Magventure) will be positioned over the upper limb motor hotspot and perpendicular to the central sulcus at a 45 °from the hemispheric midline (44) (Figure 3). The intensity for rTMS sessions will remain consistent throughout time and treatment arms.

### Sham-rTMS

In control group C, Sham rTMS will be delivered with the coil angle rotated 90° and only one wing of the coil touching the scalp of the participant to avoid inducing actual stimulation. The sound emitted during stimulation, the time and frequency of the sham rTMS will be the same as intervention group A and B (43).

### Outcomes

Participants will be evaluated by physical therapists unaware of group assignments at various time intervals using the assessments detailed in Table 1. Furthermore, baseline age, gender, symptoms, duration, prior therapy, and frequently utilized medication will be documented via a questionnaire. The primary endpoint is the four-week duration, during which any side effects will be documented in real time.

### Clinical effectiveness

#### Primary outcome measures

The CMS is a typical shoulder function metric. Effective CMS implementation has shown its validity, reliability, and responsiveness in shoulder pathology identification (45). The minimal clinically important difference (MCID) considered for the score is 17 points (46). The CMS scale evaluates four dimensions associated with shoulder pathology: two subjective factors—pain and activities of daily living (ADL)—and two objective factors—ROM and strength. The CMS employs the presence or absence of “unaffected sleep” as a criterion for scoring shoulder function (47). The subjective components can receive up to 35 points and the objective 65, culminating in a total possible score of 100 points, with higher values indicating superior functionality (48).

### Secondary outcome measures

#### Polysomnography

**Figure 4:**
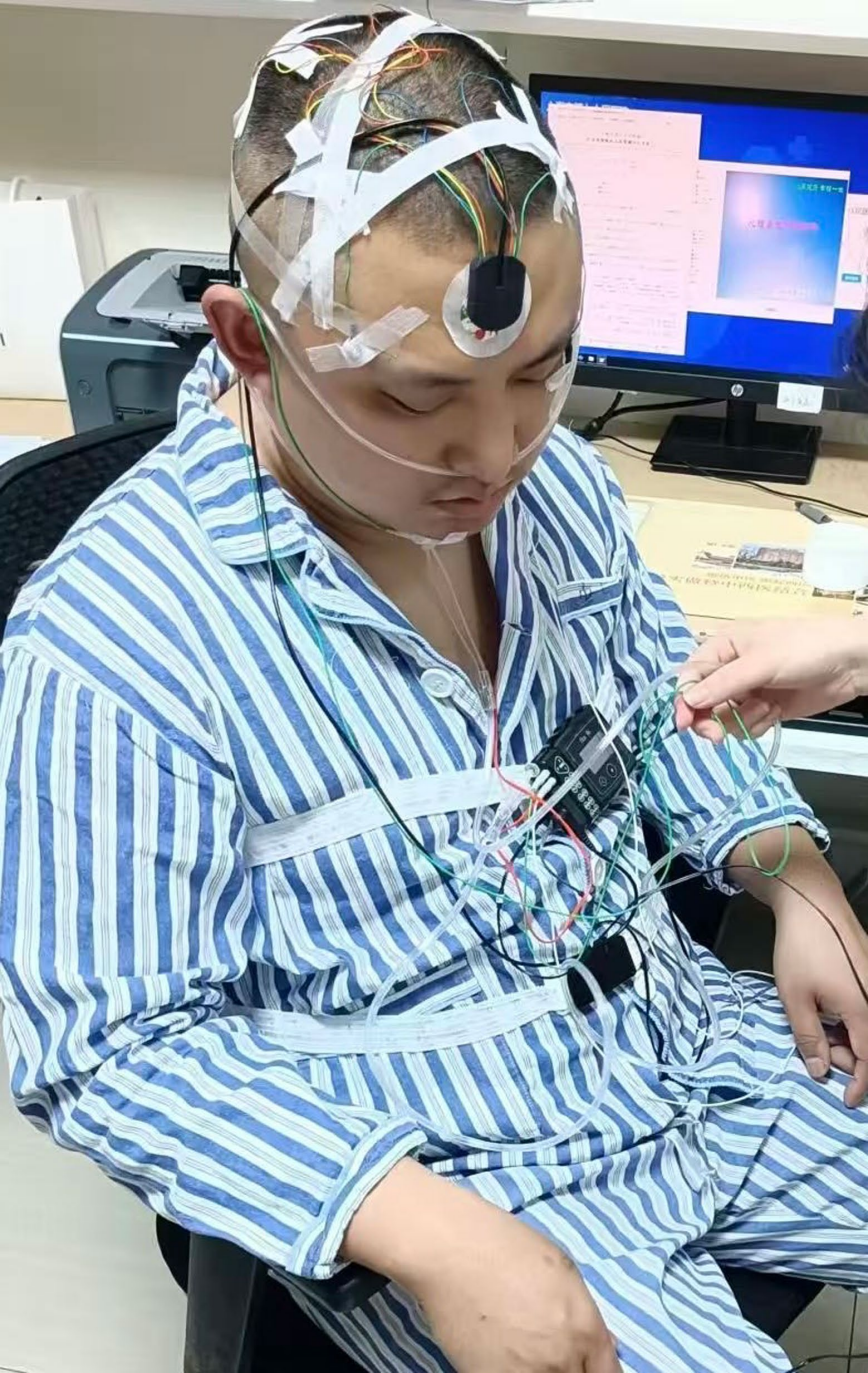
Polysomnography were performed on FS patients.

Despite a robust correlation between sleep disruption and pain, shoulder complaints and sleep disturbances have been studied little. The patients will undergo single-night polysomnography examination in the special ward in rehabilitation medicine center in Seventh People’s Hospital of Shanghai University of Traditional Chinese Medicine using a Nox A1s™ (NoX Medical, Reykjavik, Iceland) device. Recording will occur between 10:00 p.m. and 6:00 a.m., depending on the patient’s preferences and sleep patterns. The polysomnographic examinations documented electroencephalography, electrocardiography, electrooculography, and electromyography recordings from the chin area and bilaterally from the masseter muscle regions, motion recording of abdominal and thoracic breathing activity, assessment of body position, and audio recording (Figure 4). A pulse oximeter (NONIN 3150 WristOx 2; Nonin Medical Inc., Plymouth, USA) will be used to record oxygen saturation and pulse, and Noxturnal™software (Nox Medical) for sleep recording and analysis will assist in data interpretation (49).

#### 36-item short-form health survey (SF-36)

The 36-Item Short-Form Health Survey (SF-36, Chinese version) serves as the secondary outcome measure, functioning as a reliable and valid health-related questionnaire that measures QOL. The of 36 questions scale measures physical and mental health. The former encompasses physical, social, physical role, and emotional role functions. The latter contains mental health, energy fatigue, pain, and general health. The SF-36 scores range from 0 to 100, with higher scores indicating better QOL over the previous month (50).

### Neuro-biomechanism

#### Surface electromyography(sEMG)

Shoulder movements and muscle selection.

The participants performed shoulder movements including flexion, extension, abduction and upper limb coordinated movement “reach-to-grasp (RTG)” as the main basic shoulder movements and basic training processes in shoulder disfunction rehabilitation and also related to the frequent motions in ADL (51). The natural dropping state of the arm (shoulder resting state) will be added for a baseline recording of EMG signals. EMG signals from 8 muscles that control the movements of the upper arm will be recorded. Muscle names and the corresponding electrode numbers are shown in Table 2.

**Table 2:** The series number of the sEMG electrodes and the name of the muscle in which they are located.

Electrode number	Muscle	Electrode number	Muscle
1	Pectoralis Major	5	Middle Deltoid
2	Upper Trapezius	6	Posterior Deltoid
3	Supraspinatus	7	Bicep
4	Anterior Deltoid	8	Triceps

These 8 muscles will be measured using the 8-channel wireless EMG measurement system (iRecorder W8, Shanghai Idea-Interaction Tech. Co. Ltd., Shanghai, China) and eConScan data acquisition system (eConScan W8 BT, Shanghai, China). The Ag-Cl gel surface electrodes (Cathay, CH3236TD)will be used. The reference electrode will be placed on the lateral epicondylitis of humerus of the affected upper limb and the ground electrode will be placed at the inferior margin of the left sternal body adjacent to the xiphoid process. The sampling frequency will be set to 1,000 Hz. The skin will be wiped with 70% alcohol before electrodes placement. Shoulder EMG data will be collected in different groups using the following paradigms (51).

**Figure 5:**
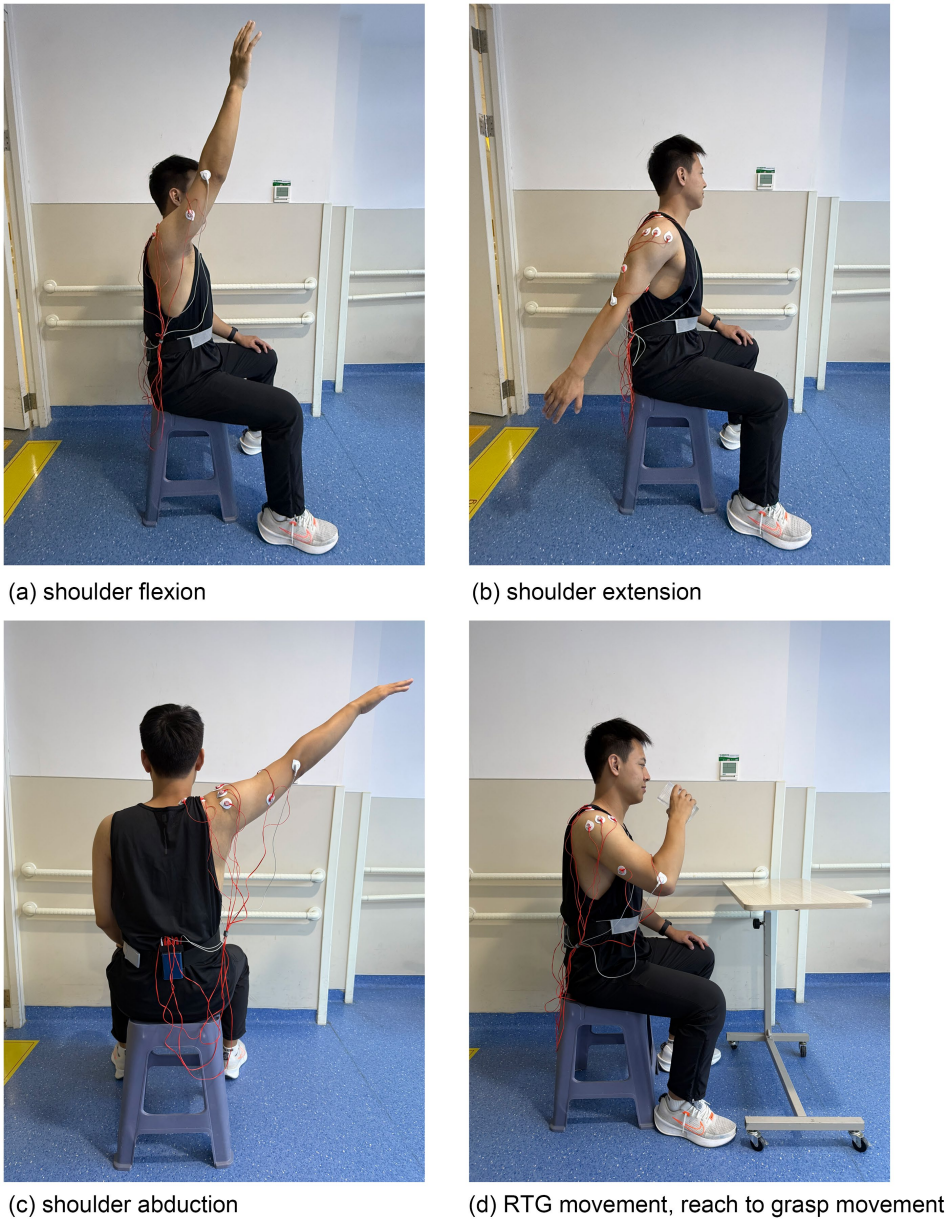
Four kinds of shoulder movements in sEMG measurement.

Paradigm 1 (Normal-speed and anti-gravity experiment): The participants will perform four kinds of shoulder movements: flexion, extension, abduction and RTG movement (drinking task with five phases: reaching to grasp a glass, forward transport of the glass to the mouth, drinking a sip of water, transporting the glass back to the table, returning the hand to the initial position) in the normal-speed without resistance just anti-gravity (Figure 5). A total of 80 EMG datasets will be collected for each participant, including 20 datasets for each movement. The action execution and collection interval are located between the two ends of the basic state interval. Resting state datasets will be collected from the baseline EMG signals for about 3 s before and after each movement. EMG baseline signal recording requires participants to relax and keep their arms at their sides.

Paradigm 2 (Normal-speed and anti-resistence experiment): The participants will wear a 5-pound wristband on the affected upper limb. The experimental platform, the actions and datasets are the same as in paradigm 1.

### Data preprocessing

The original EMG signals collected by the EMG signal acquisition platform will be 8 channel EMG signals with a sampling frequency of 1,000 Hz. In order to obtain effective information in the EMG signals, and to filter out noise and artifacts, a band pass filter of 1 − 100hz will be selected. Due to the small value of the original EMG singal voltage collected, in which the order to reduce the loss value at the beginning of the iteration, we enlarged the original data by 1,000 times.

### Motor evoked potential (MEP)

The peak-to-peak amplitude of the MEP of the FDI will be quantified as an indicator of motor evoked potential, indicating that magnetic stimulation over M1 (43) induces excitability in the descending route and contralateral muscle contraction of the recorded value. The M1 hotspot on the contralesional side of the FDI muscle will record 10 averaged MEPs at 120% of the baseline resting motor threshold. The identical stimulator output will be utilized for ensuing assessment sessions (52).

### Magnetic resonance imaging (MRI)

The MRI scan will utilize a 3.0-T GE scanner (Siemens Healthcare, Erlangen, Germany) equipped with an eight-channel phased-array head coil at the Department of Radiology of Seventh People’s Hospital of Shanghai University of Traditional Chinese Medicine, China. Each participant will get two independent brain MRI scans by two radiologists at baseline, after the 4-week intervention, and at the 6-month follow-up. The MRI will need participants to stay awake with their eyes closed.

Resting-state functional MRI (Rs-fMRI) images will be obtained using the subsequent parameters: TR = 2,100 ms, TE = 30 ms, flip angle = 90°, voxel dimensions = 3.125 mm × 3.125 mm × 3.6 mm, 42 axial slices, field of view (FOV) = 200 mm × 200 mm, and 230 phases. A 3D-BRAVO sequence with the following parameters will acquire high-resolution T1-weighted structural images (T1WI): TR = 8.2 ms, TE = 3.2 ms, flip angle = 12°, FOV = 220 mm × 20 mm, matrix = 256 × 256, slice thickness = 1 mm. The MRI results encompass GM density, WM, subcortical nuclei volumes, cortical thickness and FC. The DPARSF (http://rfmri.org/DPARSF) will preprocess fMRI data (53), while the FSL 5.0 (FMRIB Software Library) will analyze T1WI structural data (54). The volumes of neo-cortical gray matter, total gray matter, and white matter will be acquired using SIENAX, a component of FSL 5.0 (55). FMRIB’s integrated registration and segmentation tool in FSL 5.0 will calculate subcortical nuclei’s normalized volumes (56). Cortical thickness will be measured via FreeSurfer.

### Data management and monitoring

The independent Data Monitoring Committee (DMC) of the Scientific Research Innovation Platform of the Seventh People’s Hospital of Shanghai University of Traditional Chinese Medicine will oversee project data administration, data analysis and monitoring. To endure privacy and reduce bias, the dataset will be stored, analysed, and archived pseudonymously.

### Safety and adverse events

The safety officer will record and report all study adverse events. The participant’s Informed Consent Form lists all study risks. All participants will complete a TMS/MRI adverse effects questionnaire after the session.

### Data analysis

This trial will implement intention-to-treat (ITT) analysis with statistical analysis by a non-evaluation and treatment researcher. Data will be documented in the CRFs. IBM SPSS will do statistical analysis (IBM Corp. Released 2013. IBM SPSS Statistics for Windows, Version 29.0. Armonk, NY: IBM Corp.). Continuous variables are presented as mean ± SD for normal distributions, or as median and inter-quartile ranges for non-normal distributions, whereas categorical variables will be reported as frequency. We will use Two-way analysis of variance with repeated measures for continuous variables that meet the requirements of a normal distribution and homogeneity of variance, or the Wilcoxon test if not. A chi-square test will be employed for categorical variables. Primary and secondary outcomes will be compared using the Pearson correlation coefficient. Data from repeated measurements will be analyzed using two-tailed multivariate analysis of variance. *p* values below 0.05 indicate statistical significance. If needed, Bonferroni correction will be used for multiple *post hoc* comparisons.

## Discussion

FS once regarded as a peripheral joint ailment musculoskeletal disorder. Professional opinions and qualitative experience, not quantitative and current research, guided treatment selection. There is no consensus on the optimal standard treatment for patients with FS regarding pain alleviation, motor function, sleep quality and QoL (32). New perspectives are desperately required.

NMES is a treatment modality, which addresses pain and dysfunction of FS not as a simple “blocker” but as a multi-target modulator. NMES activates large-diameter, non-nociceptive sensory fibers (Aβ fibers). This increased activity “closes the gate” at the spinal cord level, thereby inhibiting the transmission of pain signals carried by small-diameter nociceptive fibers (Aδ and C fibers) to the brain. This provides immediate, short-term pain relief. Also, NMES can stimulate the release of the body’s natural pain-relieving chemicals, such as endorphins and enkephalins and activate the brain’s descending inhibitory pathways, which send signals down the spinal cord to suppress pain processing (57, 58). Pain and disuse of the shoulder joint often led to protective muscle guarding, spasm, and ischemia. NMES can break this cycle by eliciting strong, rhythmic muscle contractions followed by periods of relaxation to reduce muscle spasm and hypertonicity, improve local blood circulation, flushing out pain-inducing metabolites (e.g., lactic acid, bradykinin) as well as decrease mechanical stress on painful joints. Chronic pain is often associated with maladaptive cortical reorganization (e.g., shrinkage of the motor cortex representing the painful area) (59). By providing synchronized sensory input and motor output, NMES re-educate the neuromuscular system to maintain or restore the normal cortical representation of the affected shoulder joint and upper limb, counteracting pain-related neuroplasticity. This mechanism, to some extent, bridges the gap between peripheral stimulation and central nervous system adaptation. The rTMS as a neuromodulation technique has come into people’s view. The rTMS directly effects on the contralateral M1 of the affected shoulder joint will facilitate the recovery of pain and motor function based on the theory of central neuroplasticity. At the cellular level, stimuli can modify the electrical state of neurons; at the neurohumoral level, stimuli can evoke neurotransmitter activity; at the network level, stimuli can alter neuronal circuits; and at the behavioral level, stimuli can result alterations in pain and function (60).

The rTMS combined with NMES can be a novel and more effective therapy for shoulder function of the FS, but few studies have examined its effectiveness and neuro-biomechanism. Based on the above problems, the authors created a thorough research scheme structure with a 4-week intervention and a 6-month follow-up to achieve three study aims. This study has several important strengths. First, it’s a novel integrated treatment method combines rTMS with different frequency and NMES interventions, based on CRP for improving shoulder function after FS. Previous studies examined rTMS, NMES and CRP separately, not in an integrated method. Second, the comprehensive intervention protocol will be evidence-based and rigorously developed based on the evidence, recommendations, theories and practice standards of the systematic review. Third, this protocol will use more systematic and comprehensive assessment outcomes based on clinical effectiveness and neuro-biomechanism of brain science, such as sEMG, MEP, and GMWM/FC/subcortical nuclei volumes cortical thickness to estimate central and peripheral pain management functional neuroplasticity. Therefore, the present study will provide a more comprehensive and systematic protocol for future FS rehabilitation randomized controlled trial studies.

We acknowledge this study’s limitations. The operator cannot be blinded during the rTMS intervention due to the menus’ visibility, making blinding impossible to control. The outcome assessors and the statistician will be blinded to the group allocation; however, detection bias may still occur during the trial. Additionally, polysomnography assessment requires to be performed by medical personnel in special ward, which may limit its promotion and application in the community and home settings. Furthermore, based on the results of a few recent studies, the pain with shoulder joint motor deficit first onset and reappear were no differences, so when including patients, we did not make a clear distinction on whether these two different types of FS will affect the intervention outcomes. In future study, we should distinguish between patients experiencing their first episode and those with recurrent cases. Additionally, given the increasing incidence of the disease and the expanding age range of affected individuals (61), it will be a wise choice to stratify enrolled patients by 10-year age intervals to enhance the precision of research.

In summary, the study protocol will demonstrate the effectiveness of rTMS with/without NMES for improving shoulder function and pain management after FS and compare the efficacy of LF-rTMS and HF-rTMS. The rTMS combined with NMES may have a potential opportunity to better improve the shoulder function of FS patients. These results of the study will provide high-quality evidence to guide the design of more effective treatment methods for FS rehabilitation.
